# Single-molecule quantum dot as a Kondo simulator

**DOI:** 10.1038/ncomms16012

**Published:** 2017-06-30

**Authors:** R. Hiraoka, E. Minamitani, R. Arafune, N. Tsukahara, S. Watanabe, M. Kawai, N. Takagi

**Affiliations:** 1 Department of Advanced Materials Science, The University of Tokyo, 5-1-5 Kashiwanoha, Kashiwa, Chiba 277-8561, Japan; 2 Department of Materials Engineering, The University of Tokyo, 7-3-1 Hongo, Bunkyo-ku, Tokyo 113-8656, Japan; 3 International Center for Materials Nanoarchitectonics (WPI-MANA), National Institute for Materials Science, 1-1 Namiki, Tsukuba, Ibaraki 304-0044, Japan

## Abstract

Structural flexibility of molecule-based systems is key to realizing the novel functionalities. Tuning the structure in the atomic scale enables us to manipulate the quantum state in the molecule-based system. Here we present the reversible Hamiltonian manipulation in a single-molecule quantum dot consisting of an iron phthalocyanine molecule attached to an Au electrode and a scanning tunnelling microscope tip. We precisely controlled the position of Fe^2+^ ion in the molecular cage by using the tip, and tuned the Kondo coupling between the molecular spins and the Au electrode. Then, we realized the crossover between the strong-coupling Kondo regime and the weak-coupling regime governed by spin–orbit interaction in the molecule. The results open an avenue to simulate low-energy quantum many-body physics and quantum phase transition through the molecular flexibility.

## Abstract (web summary)

Tuning the structure in the atomic scale enables manipulation of the quantum state in a molecular based system. Here, Hiraoka *et al*. tune the Kondo coupling between molecular spins and the Au electrode by controlling the position of Fe^2+^ ions in the molecular cage with a tip.

The Kondo effect, a many-body quantum phenomenon emerging ubiquitously in various condensed matters^1^,^2^,^3^,^4^,^5^,^6^,^7^,^8^,^9^,^10^,^11^,^12^,^13^,^14^,^15^,^16^,^17^, arises from the exchange coupling of an impurity spin localized in a host with the conduction electrons. A many-body singlet, Kondo resonance, forms at the Fermi level (*E*_F_) below the Kondo temperature (*T*_K_). Spin–orbit interaction (SOI) at the impurity spin gets entangled with the Kondo effect, resulting the exotic physics^5^,^12^,^17^,^18^,^19^. The SOI lifts the degeneracy in spin quantum number *S*_z_ into multiple fine structures (denoted as SOI-splitting), causing magnetic anisotropy (MA). The SOI-splitting is often described as a uniaxial MA term of *D*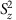, where *D* represents the splitting energy. In half-integer impurity spin with *D*>0, since the Kramers doublet of *S*_*z*_=±1/2 is the ground state, the Kondo resonance forms and coexists with the inelastic steps derived from the SOI-splitting as revealed by scanning tunnelling microscopy (STM) studies^12^,^17^. In contrast, the SOI competes the Kondo effect in integer impurity spin. For instance, in *S*=1 system of *D*>0, the SOI lifts the degeneracy into the *S*_*z*_=0 ground state and the Kramers doublet of *S*_*z*_=±1, which hampers the Kondo effect. To reveal the competition, tuning the competing strengths is indispensable. This requires the continuous control of the geometric structure around the impurity in atomic scale that is experimentally challenging.

A molecular quantum dot consisting of a single iron phthalocyanine (FePc) molecule attached to a Au(111) electrode and an STM tip serving as a counter electrode offers a good opportunity to reveal the competition. Bulk FePc molecules take *S*=1 spin triplet with the in-plane MA derived from the SOI of the Fe^2+^ ion^20^,^21^,^22^. When attached to Au(111), two unpaired electrons, one in the 3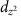 orbital and the other in the degenerate 3*d*_*zx*_/3*d*_*yz*_ (*d*_*π*_) orbitals, are Kondo-screened with the Au electrons to generate two Kondo resonances of high *T*_K_∼100 K and low *T*_K_∼2.7 K, respectively^16^. Since the bulk FePc exhibits the in-plane MA, it is expected that the competition between the Kondo effect and the SOI is hidden behind this two-spin and two-channel Kondo system. In addition, the Fe^2+^ ion in the organic cage is movable perpendicularly to the molecular plane as implied from the structural variation of haem protein molecules in which an Fe^2+^ ion in a porphyrin ring moves on the attachment and detachment of an oxygen molecule^23^,^24^. Thus, tuning the local structure around the Fe^2+^ ion in FePc can bring about a transition between the Kondo- and SOI-dominant regimes.

In this work, we experimentally and theoretically investigate the spectral evolution arising from the competition between the Kondo effect and the SOI in an *S*=1 Kondo system of FePc on Au(111). By controlling the distance between the Fe^2+^ ion and the Au substrate through approaching an STM tip to the ion, we tune the Kondo couplings over the SOI, and demonstrate the spectral crossover from the Kondo- to SOI-dominant regimes and vice versa.

## Results

### Reversible spectral evolution with STM manipulation

**Figure 1:**
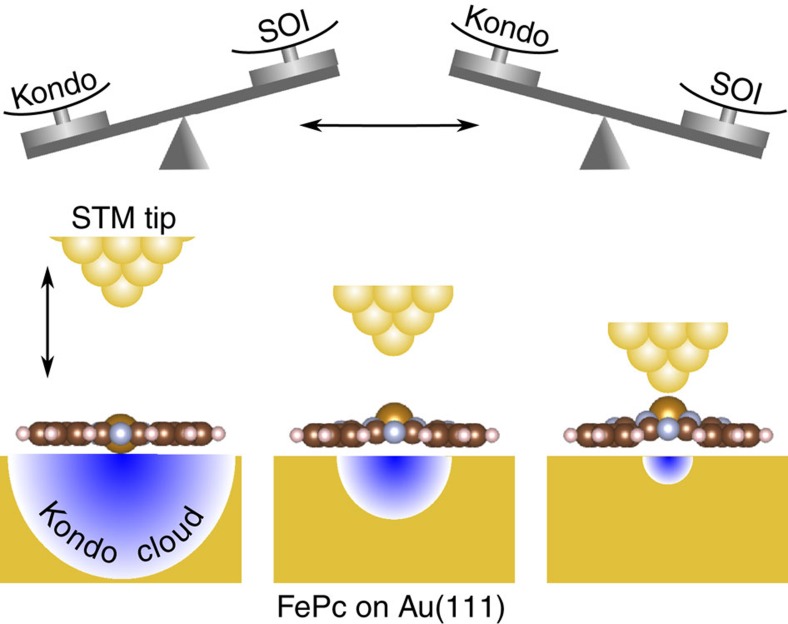
Basic concept to simulate the competition. In a molecular quantum dot consisting of an FePc molecule, an Au(111) electrode and an STM tip, controlling the STM tip position relative to the Fe^2+^ ion changes the vertical position of the ion with respect to the organic cage and distorts the molecule to a pyramidal form. The upward movement elongates the distance between the ion and the Au atom underneath, decreasing the hybridization of the 3*d* orbitals with the electrode electronic system. Controlling the movement with the STM tip enables us to tune the Kondo exchange coupling and simulate the competition. Structure model of FePc is constructed by VESTA^41^.

**Figure 2:**
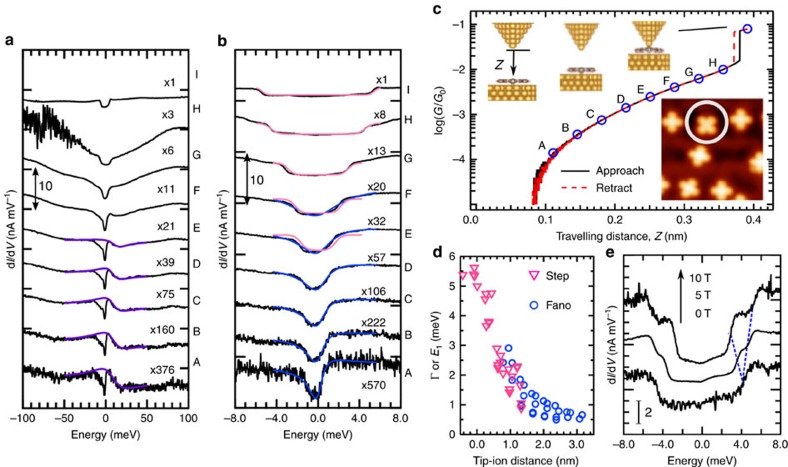
Experimental Kondo simulation with approaching an STM tip. (**a**,**b**) Evolutions of tunnelling spectra in wide and narrow energy ranges measured at 0.4 K with approaching the STM tip to the Fe^2+^ ion. The tip positions where we measured the spectra are specified with letters A–I in **c**. The modulation voltages of (**a**) 1 mV_r.m.s._ and (**b**) 0.1 mV_r.m.s._ at 312.6 Hz were added to sample voltage for the lock-in measurements. Purple and blue curves over the spectra in **a** A–E and **b** A–F are calculated with Fano function^25^, respectively. Red curves over the spectra E–I in **b** are calculated with a step function^26^. (**c**) Conductance (*G*) trace measured at 10 mV as a function of the travelling distance of the tip (*Z*). *G*_0_ is the conductance quantum. The conductance jump at *Z*=0.39 nm indicates the contact of the tip and the molecule. The inset shows an STM image (*I*=100 pA, *V*=100 mV and size of 10 × 10 nm^2^) of the target molecule marked by a circle. Structure model in the inset is constructed by VESTA^41^. (**d**) Transition of spectral feature from FK type to a symmetric step structure. Variations of Γ and *E*_1_ are plotted with the tip-ion distance, where Γ and *E*_1_ are the half width of the FK resonance and the step energy, respectively. (**e**) Spectral evolution of a step with increasing magnetic fields perpendicular to the molecular plane. These spectra were measured at 0.4 K in the configuration where the tip touches the ion. The modulation voltage of 0.2 mV_r.m.s._ at 312.6 Hz was used for the lock-in measurement. Each spectrum is vertically offset for clarity. Dotted lines guide the variation of step energy.

Figure 1 shows a concept to explore the competition between the Kondo effect and the SOI in FePc on Au(111). Approaching an STM tip to the Fe^2+^ ion in the organic cage of FePc, the ion is moved vertically and the molecule deforms to a pyramidal structure. The distance between the Fe^2+^ ion and the Au atom underneath gets longer so that the hybridization of the ion with the Au atom is reduced, resulting in the decrease of Kondo coupling relative to the SOI. Through this STM molecular manipulation, the crossover between the Kondo- and SOI-dominant regimes takes place. Figure 2a shows a series of the tunnelling spectra measured with approaching the tip to the Fe^2+^ ion. We measured the conductance as a function of the travelling distance of the tip (*Z*) (Fig. 2c) and precisely controlled the distance between the tip and the ion. The tip-ion distance is defined as (0.39−*Z*) nm from the conductance trace showing that the tip touches the ion at *Z*=0.39 nm. We confirmed that the spectral evolution is reversible. When the tip is apart from the ion (spectrum A), a broad peak and an asymmetric sharp dip appear around *E*_F_. These are the Kondo resonances of high and low *T*_K_, respectively^16^. When the tip approaches to the ion, the broad peak gradually decreases in intensity as demonstrated by the purple curves calculated with the Fano function^25^ (spectra A–E) and disappears in the spectrum F. Further approaching, a symmetric step structure appears in the spectra G–I.

Figure 2b shows the detailed spectral evolution around *E*_F_. Parallel to the decay of the broad peak, the sharp asymmetric dip in the spectrum A changes to a symmetric dip with gradual increase of the width from the spectrum B to E. The symmetric dip evolves to a step-like concave with further approaching the tip to the ion (spectra F–I). The width of the asymmetric Fano-Kondo (FK) dip is ∼1 mV in the spectrum A, which finally evolves to a pair of steps at ±5.0 mV in the spectrum I. Figure 2d shows the transition of the spectral shape. We fitted each spectrum with Fano and step functions^26^ and determined which function better reproduces the spectrum (details are given in Supplementary Note 1). When the tip-ion distance ranges from 0.3 to 0.2 nm, the Fano function well reproduces the spectra while a step function better explains the spectra for 0–0.1 nm. The transition continuously undergoes from the FK dip to the step-function form. The step splits by applying magnetic fields (Fig. 2e). The splitting matches the spin-Zeeman effect in the Kramers doublet of *S*_*z*_=±1 (See Supplementary Note 2), which means that the step originates from the inelastic excitation between the lower state of *S*_*z*_=0 and the upper Kramers doublet derived from the SOI-splitting. Combined with the theoretical analysis below, the measured spectral evolution is attributed to the crossover from the Kondo regime to the SOI regime. The approaching tip moves the ion upward and reduces the Kondo couplings. The decrease of the Kondo couplings lowers both *T*_K_’s to suppress the Kondo screening; consequently the SOI-splitting becomes dominant, leading the inelastic step structure.

### Variation in geometric and electronic structures of FePc

**Figure 3:**
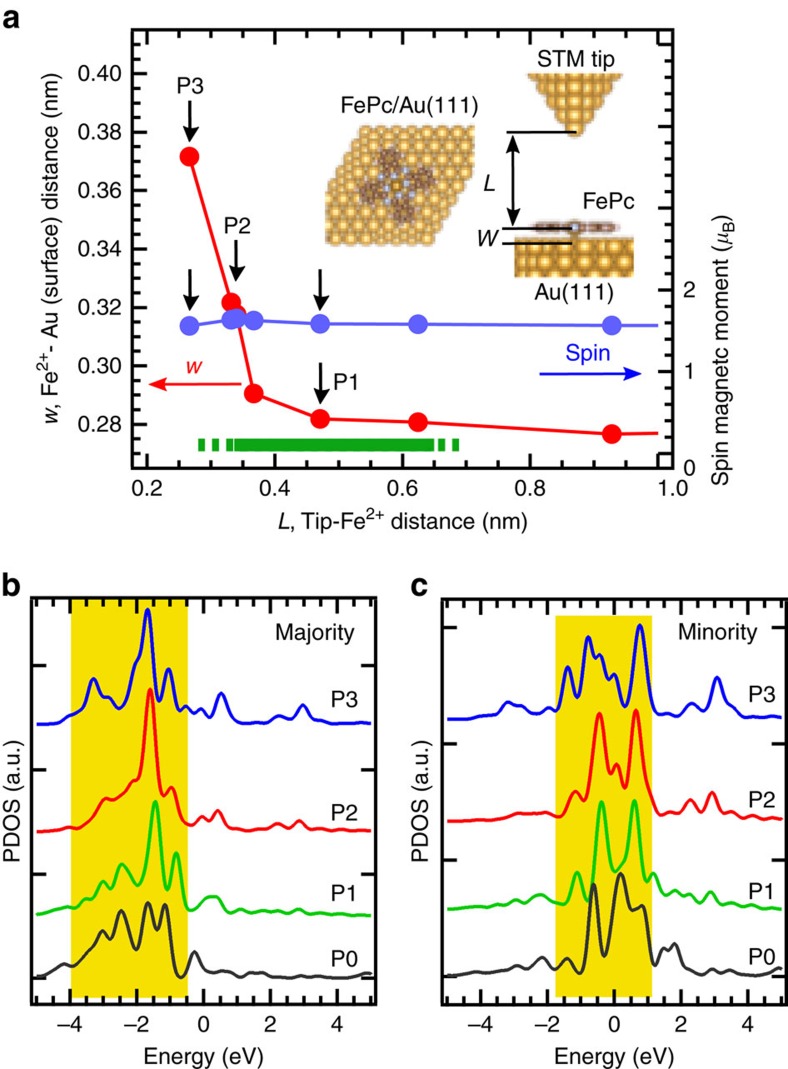
Variation of geometric and electronic structures. (**a**) Variation of distance between the Fe^2+^ ion and the Au atom underneath (defined as *w*) and the spin magnetic moment in Fe 3*d* orbitals as a function of the distance between the ion and an apex Au atom of approaching STM tip (defined as *L*). The inset shows the calculated structure models rendered by VESTA^41^. FePc is located at the on-top site of Au(111). Green horizontal bar indicates the travelling range of tip in the STM experiment. (**b**,**c**) Variation of PDOS spectra of 3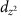 for the **b** majority and **c** minority spins as a function of tip-molecule configuration (P0–P3). P0 represents the configuration without the tip. P1–P3 indicate the tip positions marked in **a** with small arrows. Highlighted regions show marked differences in the PDOS spectra.

We demonstrate using density functional theory (DFT) calculations that the position of the Fe^2+^ ion is controllable with the STM tip and that the movement of the ion varies the hybridization of the 3*d* orbitals with the substrate. Figure 3a shows the distance of the ion with the substrate Au atom underneath (*w*) and the spin magnetic moment (*M*_S_) as a function of the distance between the ion and the tip (*L*). Without the tip (P0), *w*=0.277 nm and *M*_S_=1.58 μ_B_ (μ_B_ is the Bohr magneton). Approaching the tip to the ion, the ion slightly moves upward with *w*=0.282 nm at P1, which extends to 0.291 nm at *L*=0.367 nm, 0.322 nm at P2 (*L*=0.331 nm) and finally 0.372 nm at P3 (*L*=0.266 nm). During the structural variation, *M*_S_ is constant and *S*=1 is preserved; the occupation of the *d* orbitals is almost unchanged.

The *L*-dependent projected density of states (PDOS) spectrum of the 3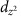 orbital shows a marked variation (Fig. 3b,c). The spectrum of the majority spin at P0 shows multiple broad peaks ranging from −1 to −4 eV. Moving the tip from P0 to P1 and P2, the peak at −1.5 eV grows in intensity to narrow while the peak at −1 eV decreases in intensity. In addition, the broad structure extending from −4 to −2 eV decreases. At P3, the peak at −1.5 eV becomes broader with the increase of the structure around −3 eV because of the hybridization with the tip states. Compared with the spectrum at P0, the overall spectral shape is still sharp, indicating that the hybridization with the tip is weaker than that with the substrate at P0. Similar variations occur in the minority spin as highlighted. These variations in both majority and minority spins indicate that the upward movement of the Fe^2+^ ion reduces the hybridization of the 3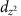 orbital with the substrate. The PDOS spectra of the other 3*d* orbitals change slightly, but their occupations are unchanged (see Supplementary Note 3).

### Numerical renormalization group analysis

**Figure 4:**
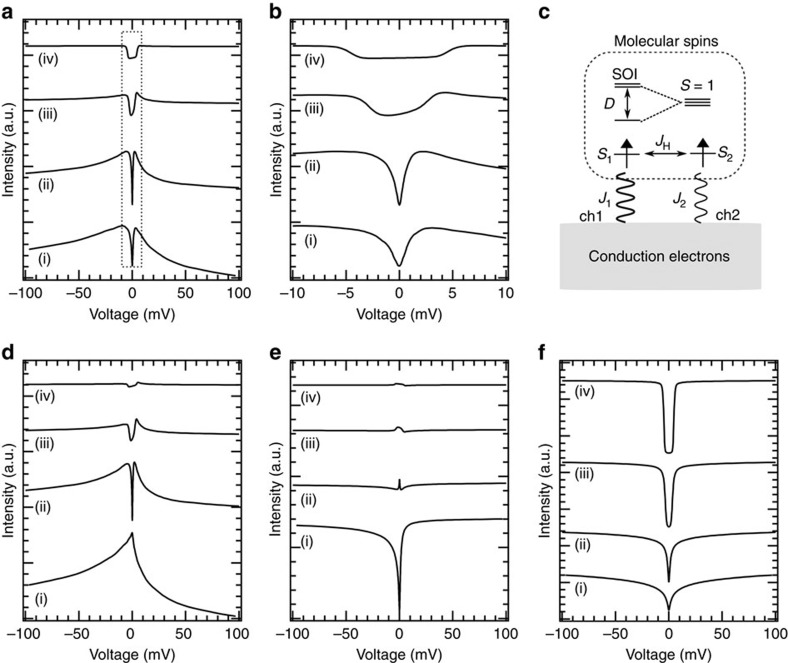
Spectral evolution calculated by NRG. (**a**,**b**) Spectral evolutions calculated with a two-orbital and two-channel Kondo model corresponding to Fig. 2a,b, respectively. The spectra marked by a dotted rectangle in **a** are shown in **b**. The NRG calculations were performed in four regimes: (i) strong-coupling regime where the Kondo effect is dominant (*J*_1_=0.75, *J*_2_=0.4 eV), (ii) medium-coupling regime 1 (*J*_1_=0.6, *J*_2_=0.32 eV), (iii) medium-coupling regime 2 (*J*_1_=0.45, *J*_2_=0.24 eV) and (iv) weak-coupling regime where the SOI is dominant (*J*_1_=0.3, *J*_2_=0.16 eV). Each spectrum in **a** was calculated by summing the FK resonance spectra shown in **d**,**e** with the inelastic spin excitation spectrum in **f**. The intensity was adjusted to reproduce the overall spectral evolution in Fig. 2a. The spectra (i) and (iv) correspond to those A and I in Fig. 2a, respectively. (**c**) Two-orbital and two-channel Kondo model. Two impurity spins in 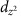 (*S*_1_) and *d*_*π*_ (*S*_2_) orbitals couple the conduction electrons through the screening channels 1 and 2 with the Kondo coupling constants of *J*_1_ and *J*_2_, respectively. These spins also couple through the Hund’s rule (*J*_H_). The channels 1 and 2 cause the Kondo resonances of high and low *T*_K_’s. The SOI-splitting is described as a uniaxial MA term of *D*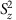. We set *D*=5 meV and *J*_H_=0.8 eV. (**d**,**e**) FK resonance spectra calculated for the channels 1 and 2 (high- and low-*T*_K_ channels), respectively. (**f**) Inelastic spin excitation spectra calculated for the regimes (i)–(iv).

To gain insights of the crossover between the Kondo- and SOI-dominant regimes, we modelled our system by a two-orbital and two-channel Kondo Hamiltonian (Fig. 4c). The Hamiltonian is described as,


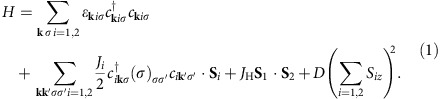


Here the first term represents the conduction electrons of the Au(111) surface. 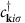 and *c*_**k***iσ*_ are creation and annihilation operators of the surface electron specified by the crystal momentum **k** and spin *σ*. The second term describes the *s*–*d* Kondo exchange couplings (denoted as *J*_1_ and *J*_2_) of the two localized spins with the surface electrons. **S**_1_ and **S**_2_ are spin in the 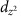 and *d*_*π*_ orbitals, respectively. *i*=1 and *i*=2 specify the 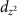 (screening channel 1) and *d*_*π*_ (screening channel 2) orbitals, respectively. The third term represents the Hund’s coupling between the two spins. We set *J*_H_ at −0.8 eV. The last term describes the SOI-splitting. We assume a uniaxial MA term of *D*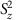. The constant *D* is the excitation energy observed experimentally from the lowest state of *S*_*z*_=0 to the doubly degenerate excited states of *S*_*z*_=±1.

The parameters *D*, *J*_1_ and *J*_2_ play a central role in our model. We consider how these parameters vary according to the position of the Fe^2+^ ion relative to the Au surface. Recent STM work for an Fe-porphyrin (FeP) derivative on Pb(111)^27^ has demonstrated that *D* can be varied through the change in the ligand field splitting caused by approaching and contacting the STM tip to the molecule. Similar variation of *D* may occur in our system, because the local structure around the Fe ion in FePc resembles that in the FeP derivative. In the STM work^27^, the manipulation with tip only reduces *D* by 10% at most, and such small reduction of *D* hardly drives the crossover between the Kondo effect and the SOI. Thus, we set *D* at the constant value of 5 meV from the measured step energy. In contrast, *J*_1_ and *J*_2_ can be largely varied. The DFT results (see Fig. 3 and Supplementary Figs 2 and 4) show that the upward movement of the ion induced by the tip decreases the hybridizations of the *d* orbitals with the substrate electrons. Consequently, *J*_1_ and *J*_2_ are reduced with the movement, leading to the lowering of respective *T*_K_’s. Thus, tuning *J*_1_ and *J*_2_ allows us to investigate the crossover. The larger (smaller) *J*_1_ and *J*_2_ result in the Kondo (SOI)-dominant regime. The details about the model and the evaluation of *J*_1_ and *J*_2_ from the DFT calculations are described in Supplementary Notes 4 and 5.

We calculated the spectral evolutions by the numerical renormalization group (NRG) calculations with equation (1). The calculation details are described in Supplementary Note 6. Figure 4a,b shows the spectral evolutions calculated for those in Fig. 2a,b, respectively. The calculated results reasonably reproduce the experimental ones, providing solid evidence that the competition between the Kondo effect and the SOI drives the crossover. The non-monotonic spectral evolution is rationalized by the combination of the variations of the FK resonance spectra in high-*T*_K_ and low-*T*_K_ channels (Fig. 4d,e, respectively) and the inelastic excitation spectrum (Fig. 4f). The asymmetric spectral shape in Fig. 4d and the dip are derived from the Fano effect.

In Fig. 4d, an asymmetric broad FK peak appears in the regime (i). The peak slightly decays and a sharp dip emerges in the regime (ii). In the regime (iii), the peak further decays, and the dip also becomes shallower while it becomes wider. Finally, the residue of the dip remains in the regime (iv). The dip comes from the gap in the DOS spectrum caused by the SOI-splitting (see Supplementary Fig. 5). The widening of the dip indicates that the SOI-splitting is hardly getting renormalized by the Kondo effect as the Kondo exchange couplings become smaller. The FK resonance in the low-*T*_K_ channel decays much faster (Fig. 4e). A sharp FK dip in the regime (i) almost disappears in the regime (ii). Thus, the contribution from the low-*T*_K_ channel to the tunnelling spectra in the medium- and weak-coupling regimes is negligible, and consequently the variation of the FK resonance in the high-*T*_K_ channel dominantly determines the spectral evolution. However, only the FK resonance from high-*T*_K_ channel can hardly explain the spectra in the regimes (iii) and (iv). In these regimes, the inelastic tunnelling process with spin excitation is dominant over the normal tunnelling and determines the spectral shapes. With approaching the tip to the ion, the crossover from the Kondo regime to the SOI regime occurs simultaneously with the switching in the two tunnelling processes responsible for the spectral shape.

## Discussion

The crossover can hardly be explained by the mixed valence model, the other candidate of the electronic ground state of FePc on Au(111) described by a mixed configuration of *d*^6^+*d*^7^ in the Fe^2+^ ion^21^. Indeed, as shown in Fig. 3, the total spin magnetic moment is slightly smaller than 2 μ_B_, and the PDOS spectra of 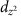 orbital indicate that the occupation exceeds 1. One might consider that the mixed valence model gives more suitable description for the electronic ground state of FePc on Au(111) than the Kondo model. We calculated the excitation spectra in the mixed valence model with a two-orbital Anderson Hamiltonian by using the NRG method (see Supplementary Note 7). In the weak SOI regime, a broad asymmetric peak appears at the Fermi level in the mixed valence model as a result of the charge fluctuation in the 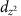 orbital (see Supplementary Fig. 9). This spectral feature is similar to the spectrum A in Fig. 2a and spectrum (i) in Fig. 4a, respectively. Hence, it is difficult to conclude which model is more suitable from the spectral shape alone. However, the spectral change in the SOI-dominant regime enables us to judge the suitable model. In the mixed valence model, the asymmetric peak due to the charge fluctuation enhances the intensity in the SOI-dominant regime because the charge fluctuation does not compete with the SOI. This contrasts to the variation of Kondo peak. As shown in Fig. 4 and Supplementary Fig. 9, the Kondo peak almost disappears in the SOI-dominant regime due to the competition. Obviously, the spectral evolution in the STM experiment does not match with that in the mixed valence model. Therefore, we conclude that the Kondo model better describes the electronic ground state of FePc on Au(111) (the detailed discussion is provided in Supplementary Note 7).

Finally, we note that the spectral variation observed in this work is essentially different from the results reported so far by similar STM junction works. For a Co atom on Cu(100)^10^, the tip approach increases *T*_K_. The increase arises from the energy shift of *d* orbital relevant to the Kondo effect. In the regime where the tip is contacted with the Co adatom, further increase of *T*_K_ is observed^15^. This enhancement is rationalized by the structural relaxation around the adatom and the resultant variation in the electronic structure. In contrast, *T*_K_ is almost unchanged for a Co atom on Cu(111)^11^. This is because the energy position of the *d* orbital does not change by approaching the tip to the adatom. These results are different from the present results. The non-equilibrium effects in high current and finite bias are unlikely to explain the spectral evolution. High tunnelling current through the STM junction can destroy the Kondo correlation due to the local heating by the electron–electron (e–e) inelastic scattering. Comparing the size of Kondo cloud and the inelastic e–e scattering length provides a measure to judge whether this heating effect is dominant^15^. The size of Kondo cloud is described as *ħv*_F_/*k*_B_*T*_K_, where *ħ* is the Planck constant divided by 2*π*, *v*_F_ is the Fermi velocity of conduction electron and *k*_B_ is the Boltzmann constant^28^. The cloud sizes are estimated to be ∼0.1 and ∼3 μm for the Kondo resonances of the localized spins in the 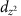 and *d*_*π*_ orbitals of FePc on Au(111), respectively. The e–e scattering length of electron in the energy range of ±10 meV with respect to the Fermi level is the order of ∼10^3^ μm (refs 29, 30) much longer than the sizes of the Kondo clouds. Therefore, the inelastic scattering is negligible and the disappearance of the Kondo resonances cannot be rationalized by the local heating effect. The effect of finite bias is observed for a Mn-porphyrin molecule on Au(111)^31^. An asymmetric Kondo peak inverts with respect to the Fermi level when the tip contacts with the Mn ion. This spectral variation cannot explain our result, reversible spectral crossover from the FK resonances to the inelastic step structure or vice versa.

The present study reveals the complex nature of the entanglement between the Kondo effect and the SOI, and also demonstrates the tunability through controlling the molecular structure with sub-angstrom resolution. Our technique provides a unique pathway to experimentally explore the intriguing physics that have been accessible only to the theoretical calculations so far, would stimulate the further theoretical development and extend to the other complicated many-body quantum physics such as the interplay between magnetism and superconductivity and quantum phase transition.

## Methods

### STM measurements

All the STM experiments were conducted in an ultra-high vacuum chamber (base pressure of 5 × 10^−11^ Torr). A Au(111) single crystal was cleaned by the repeated cycles of Ar^+^ ion sputtering and annealing. FePc molecules were deposited by heating a home-built cell at 560 K onto the Au(111) substrate kept at room temperature. An STM tip was fabricated by electrochemically etching a W wire. The shape of the tip apex was checked with field ion microscopy and tuned by gently touching the tip to the clean Au(111) surface. As a result, the apex of STM tip was coated with Au atoms. The differential conductance (d*I/*d*V*) spectra were measured with a lock-in technique; the modulation voltage was 0.1–1 mV_r.m.s._ at the frequency of 312.6 Hz. The molecular junction was constructed by attaching the STM tip to the FePc molecule on the substrate. The conductance traces were measured as a function of the vertical travelling distance (*Z*) of the tip relative to the position when the feedback was turned off. The magnetic field was applied perpendicularly to the surface up to 10 T by using a superconducting magnet.

### DFT calculations

DFT calculations were made with the Vienna ab initio simulation package^32^,^33^ with the projected augmented wave method^34^. Exchange and correlation were described at the level of local density approximation^35^,^36^. For the *d* electrons in the Fe^2+^ ion, we have employed the local density approximation+U method^37^ with *U*=2.0 and *J*=1.0 eV. The adsorbed FePc on Au(111) was modelled by (8 × 8) supercell, which consists of an FePc on a four-layer Au slab with a vacuum of ∼1.56 nm thick along the surface normal. The STM tip was composed of Au atoms because the tip apex was coated with Au atoms by gently touching the W tip to the Au(111) surface in the STM experiments. The atoms in the bottommost Au layer were fixed at their ideal bulk positions during the structure optimization.

### NRG calculations

The NRG calculations were carried out by our own built simulation code. We use the logarithmic discretization parameter *Λ*=3.5 and the number of the kept low-lying eigenstates is around 2,500–2,600 states in respective iterations. We utilize the z-averaging technique^38^ with number of z-parameter *N*_*z*_=10 to remove the artificial oscillation in the calculated spectrum. In the calculation of the elastic/inelastic excitation spectrum, we implemented the method by Anders *et al*.^39^,^40^ in which the NRG data from all the iterations are combined to construct a complete Fock space to obtain highly accurate excitation spectrum. All the NRG calculations were performed at 0 K in the present work.

### Data availability

The data that support the findings of this study are available from the corresponding authors on request.

## Additional information

**How to cite this article:** Hiraoka, R. *et al*. Single-molecule quantum dot as a Kondo simulator. *Nat. Commun.* **8,** 16012 doi: 10.1038/ncomms16012 (2017).

**Publisher’s note**: Springer Nature remains neutral with regard to jurisdictional claims in published maps and institutional affiliations.

## Supplementary Material

Supplementary Information
