# Supplementary material for: Single-molecule quantum dot as a Kondo simulator
Source: Nat Commun. 2017 Jun 30;8:16012. doi: 10.1038/ncomms16012 (PMC5497065; doi:10.1038/ncomms16012)
Supplement: Supplementary Information [file ncomms16012-s1.pdf]

File name: Supplementary Information
Description: Supplementary figures, supplementary tables, supplementary notes and supplementary references.

Supplementary Note 1 | Analysis of tunneling spectra

Tunneling spectra in Figs. 2a and 2b were fitted with two types of spectral functions; one is Fano function[1] for the Kondo signature and the other a step function[2] for the inelastic excitation structure. The Fano and step functions are defined as

$$f_{\mathrm{Fano}}(eV) = A_1 \frac{(\varepsilon+q)^2}{1+\varepsilon^2} + A_2, \varepsilon = \frac{eV-\varepsilon_0}{\Gamma}, \quad (1)$$

and

$$f_{\mathrm{step}}(eV) = B_1 \left[F\left(\frac{-eV+E_1}{k_{\mathrm{B}}T_{\mathrm{eff}}}\right) + F\left(\frac{eV+E_1}{k_{\mathrm{B}}T_{\mathrm{eff}}}\right)\right] + B_2, F(x) = \frac{1+(x-1)e^x}{(e^x-1)^2}, \quad (2)$$

respectively. Here, q is the Fano asymmetric parameter, ε_0 is the energy of the Kondo resonance, and Γ is the half width of the Kondo resonance. For the step function, E_1 represents an excitation energy associated with the inelastic step, k_{B} is the Boltzmann constant, and T_{eff} is an effective temperature which includes both extrinsic and intrinsic broadenings. In addition, the constants A_1 and A_2 (B_1 and B_2) are the coefficients to fit the spectral intensity and the background, respectively.

These spectral functions are derived independently from the different physical origins; Supplementary Eq. (1) originates from the Fano interference between two tunneling channels while Supplementary Eq. (2) is a phenomenological spectral function to describe the step structure caused by the inelastic electron tunneling. Thus, it is physically meaningless to compare A_1 and B_1 for discussing the crossover between the Kondo- and SOI-dominant regimes. Instead, we discussed the crossover based on the residual errors in the spectral fitting. From the residual errors, we determined which spectral functions better reproduce the measured spectra, and then plotted the results with the pink and blue markers in Fig. 2d. Supplementary Table 1 shows the residual errors for the series of spectra in Fig. 2b. As shown in Fig. 2b and Supplementary Table 1, the spectra A to D are reproduced with the Fano function while the spectra G-I are fitted better with the step function. The spectra E and F can be fitted by both functions. The residual errors for the spectral fitting with both functions are almost the same so that we cannot determine which is better, indicating the transition between the Kondo- and SOI-dominant regimes. All the spectra measured in the narrow energy range were analyzed in this way and then we plotted Γ and E_1 as a function of the tip-molecule distance as shown in Fig. 2d.

The spectra measured in the wide energy range (Fig. 2a) were also fitted with the Fano function to evaluate the evolution of the high-T_{K} Kondo resonance. The purple curves in Fig. 2a are the calculated results. A broad peak structure around E_{F} is well reproduced in each spectrum from A to E. The peak structure gradually reduces in intensity and almost disappears in the spectrum F. We were not able to fit the spectra of F-I with the Fano function. We used q = -1.12 and 0.38 for the spectra A in Figs. 2a and 2b, respectively.

Supplementary Table 1 | Comparison of spectral fitting with two spectral functions. The measured spectra are fitted with Supplementary Eqs. (1) and (2), and the residual errors are listed. X indicates that the fitting is not done successfully because the spectral shape is far different from the spectral function. The errors for the spectra A and B with Supplementary Eq. (1) are larger than the others despite that the spectra are well reproduced. This is mainly because the noise levels are higher compared to the others.

Spectrum (Fig. 2b)	Error with Supplementary Eq. (1)	Error with Supplementary Eq. (2)
A	6.96	X
B	3.42	X
C	2.13	X
D	1.76	X
E	1.87	5.05
F	2.09	3.36
G	X	1.64
H	X	1.07
I	X	1.58

Supplementary Note 2 | Zeeman splitting of inelastic step

To reveal the origin of the step structure, we measured the spectral response to external magnetic fields. Supplementary Figure 1a shows the spectral variation measured by increasing the magnetic field from 0 to 10 T. Supplementary Figure 1b shows the energies of split steps as a function of magnetic field. The variations of these steps are well described with spin Zeeman effect of $S_z = \pm 1$. The least square fitting yields the Lande g-factor of 2.0$\pm$0.4. These results clearly indicate that the SOI partially splits the spin triplet into the spin substate of $S_z = 0$ and the doublet of $S_z = \pm 1$. The tunneling electron excites a transition between the two substates, and thus a step structure appears.

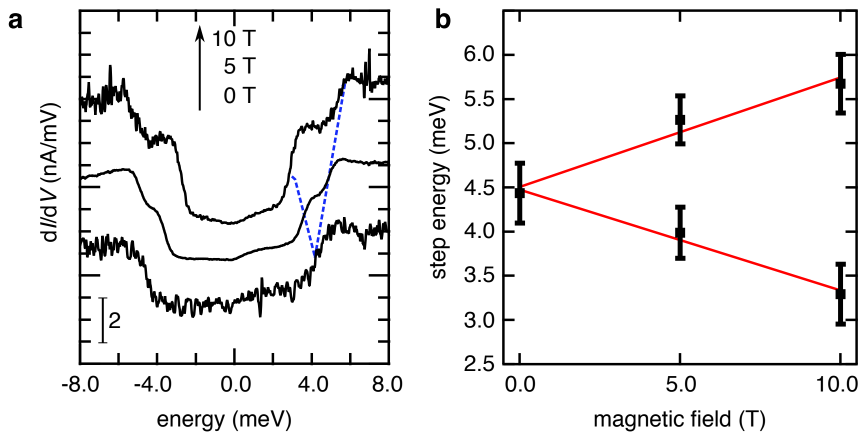

Supplementary Figure 1 | Magnetic field evolution of the inelastic step. (**a**) Spectral variation of a step with increasing magnetic fields perpendicular to the molecular plane. These spectra are measured at 0.4 K in the configuration where the tip touches the ion. The modulation voltage of 0.2 mV_{rms} at 312.6 Hz is used for the lock-in measurement. Each spectrum is vertically offset for clarity. Dotted lines guide the variation of step energy. (**b**) Variation of the step energies as a function of the magnetic field. The error bars at 0, 5, 10 T represent the standard deviations determined from 14, 28 and 25 counts, respectively. The linear lines are the results of least-square fitting, yielding Lande g-factor of 2.0±0.4.

Supplementary Note 3 | Variation in PDOS of 3d orbitals and charge distribution

The variations of PDOS spectra of $3d_{zx}(3d_{yz})$, $3d_{xy}$, $3d_{x^2-y^2}$, and $3d_{z^2}$ orbitals are shown in Supplementary Fig. 2. Although the PDOS spectra of $3d_{zx}(3d_{yz})$, $3d_{xy}$ and $3d_{x^2-y^2}$ orbitals are slightly changed, the positions of the dominant peaks do not shift seriously and thus their occupations are basically unchanged. The degeneracy in the $3d_{zx}$ and $3d_{yz}$orbitals is preserved.

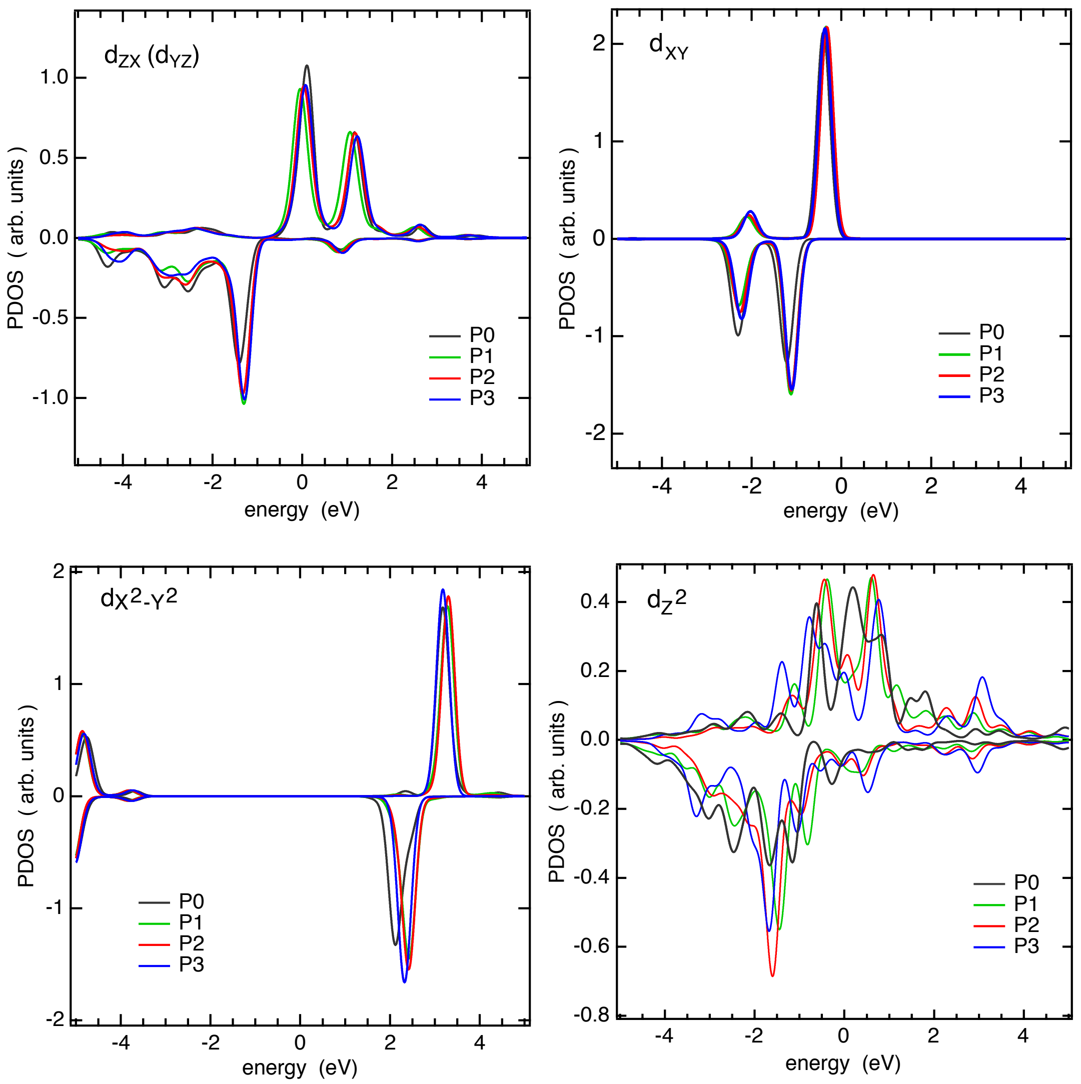

Supplementary Figure 2 | Variations of PDOS spectra of 3*d* orbitals. The PDOS spectra of $3d_{zx}$ ($3d_{yz}$), $3d_{xy}$, $3d_{x^2-y^2}$, and $3d_{z^2}$ orbitals are shown as a function of tip-molecule configuration.

Supplementary Figure 3 shows the differential charge distribution calculated for the P2 configuration. The charges distribute not only in the interface region between FePc and Au(111) but also in the region between the Fe^{2+} ion and the apex atom of tip. The differential charge distribution $\Delta\rho(\boldsymbol{r})$ is defined as

$$\Delta\rho(\boldsymbol{r}) = \rho_0(\boldsymbol{r}) - \rho_{\mathrm{mol}}(\boldsymbol{r}) - \rho_{\mathrm{Au(111)}}(\boldsymbol{r}) - \rho_{\mathrm{tip}}(\boldsymbol{r}), \quad (3)$$

where $\rho_0(\boldsymbol{r})$ is the charge distribution of a molecular quantum dot consisting of FePc on Au(111) and the STM tip, $\rho_{\mathrm{mol}}(\boldsymbol{r})$ represents that of FePc, and $\rho_{\mathrm{Au(111)}}(\boldsymbol{r})$ and $\rho_{\mathrm{tip}}(\boldsymbol{r})$ represent those of the Au(111) substrate and the STM tip, respectively. First, the total geometric structure of the molecular quantum dot is optimized. Then, $\rho_0(\boldsymbol{r})$, $\rho_{\mathrm{mol}}(\boldsymbol{r})$, $\rho_{\mathrm{Au(111)}}(\boldsymbol{r})$ and $\rho_{\mathrm{tip}}(\boldsymbol{r})$ are calculated by using the optimized structure parameters. Finally, the differential charge distribution is calculated by using Supplementary Eq.

(3).

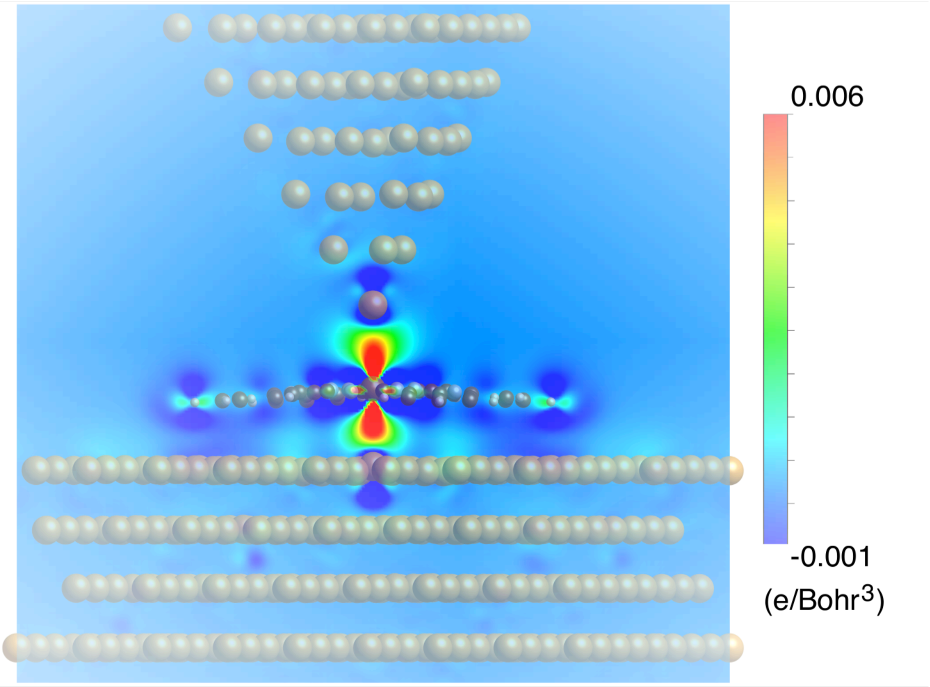

Supplementary Figure 3 | Differential charge distribution. The cross-sectional view of differential charge distribution cut by a plane including the Fe^{2+} ion is shown. The color bar represents the charge accumulation (red) and depletion (blue). The FePc molecule is slightly deformed into a pyramidal form by the interaction between the ion and the STM tip.

Supplementary Note 4 | Two-orbital and two-channel Kondo model

In this section, we describe the two-orbital and two-channel Kondo model discussed in the main text. The present DFT calculations show two localized spins, one in the d_{z^2} orbital and the other in the degenerate d_π orbitals, in the electronic ground state of FePc on Au(111). These localized spins interact with the surface electrons and form the Kondo resonance states, respectively, when the tip is far away from the molecule. As the tip approaches to the molecule, the spectrum shows the crossover from the FK resonances to the inelastic step structure. This situation is well described by the following Hamiltonian,

$$H = H_{\mathrm{K}} + H_1, \qquad (4)$$

$$H_{\mathrm{K}} = \sum_{\mathbf{k}\,\sigma\,i=1,2} \varepsilon_{\mathbf{k}i\sigma} c^{\dagger}_{\mathbf{k}i\sigma} c_{\mathbf{k}i\sigma} + \sum_{\mathbf{k}\,\mathbf{k}'\,\sigma\,\sigma'\,i=1,2} \frac{J_i}{2} c^{\dagger}_{i\mathbf{k}\sigma} (\boldsymbol{\sigma})_{\sigma\sigma'} c_{i\mathbf{k}'\sigma'} \cdot \mathbf{S}_i \,, (4a)$$

$$H_1 = J_{\mathrm{H}} \mathbf{S}_1 \cdot \mathbf{S}_2 + D \left(\sum_{i=1,2} S_{iz} \right)^2 . \ (4b)$$

Here, H_{K} represents the Kondo Hamiltonian with two localized spins interacting with the surface electrons. The first term represents the conduction electrons of the Au(111) surface. $c^{\dagger}_{\mathbf{k}i\sigma}$ and $c_{\mathbf{k}i\sigma}$ are creation and annihilation operators of the surface electron specified by the crystal momentum $\mathbf{k}$ and spin σ. The second term describes the *s-d* Kondo exchange couplings (denoted as J_1 and J_2) of the two localized spins (denoted as $\mathbf{S}_1$ and $\mathbf{S}_2$) with the surface electrons. $\mathbf{S}_1$ and $\mathbf{S}_2$ are spin in the d_{z^2} and d_π orbitals, respectively. The degeneracy in the d_π orbitals is neglected for simplicity. $i = 1$ and $i = 2$ specify the d_{z^2} (screening channel 1) and d_π (screening channel 2) orbitals, respectively. The first term of H_1 represents the Hund's coupling between the two spins. We set J_{H} at -0.8 eV. The second term describes the SOI-splitting. We assume a uniaxial MA term of DS_z^2. The constant D is the excitation energy from the lowest state of $S_z = 0$ to the doubly-degenerate excited states of $S_z = \pm 1$. We set D at 5 meV from the spectrum I in Fig. 2. H_{K} and H_1 compete each other. In this model, the total charge per respective channels and total S_z are conserved.

The parameters J_1 and J_2 govern the spectral evolution. When approaching the STM tip to the molecule, the Fe ion moves gradually upward as demonstrated by the DFT calculations. This movement reduces the hybridizations of the relevant *d* orbitals with the surface electrons and decreases the values of J_1 and J_2, lowering the Kondo temperatures and leading to the crossover between the two regimes. Therefore, we calculated the spectral evolution by treating J_1 and J_2 as model parameters with fixing J_{H} and D, and qualitatively compared the calculated and the measured spectra.

The decrease of J_1 and J_2 during tip approaching discussed above can be confirmed by the DFT calculation qualitatively. Supplementary Figure 4 shows the PDOS spectra of d_{z^2} orbital calculated for

FePc on Au(111) as a function of the molecular configuration (P0, P2 and P3 in Fig. 3a). In order to extract the variation in the hybridization of the *d* orbital with the surface and remove the hybridization with the tip, we calculated the PDOS spectra by using the model structure in which the structure of FePc on Au(111) is fixed in the same as Fig. 3a without the tip. In the majority-spin PDOS of P0, multiple peaks extend from -5 to -1 eV as a result of the hybridization with the substrate electronic states (Supplementary Fig. 4a). These peaks merge to a main peak around -2 eV from P0 to P2 and P3. Overall spectral width obviously becomes narrower from P0 to P3. Similar spectral variation is observed in the minority-spin PDOS as shown in Supplementary Fig. 4b. These spectral variations in both majority- and minority-spin PDOS indicate the decrease of the hybridization of the d_{z^2} orbital with the substrate electrons along with the upward movement of Fe ion and consequently decrease of J_1 and J_2 which are proportional to the hybridization. We confirm that the PDOS spectra are almost the same for U = 1.5-3.0 eV in the LDA+U calculations and do not depend strongly on the choice of U.

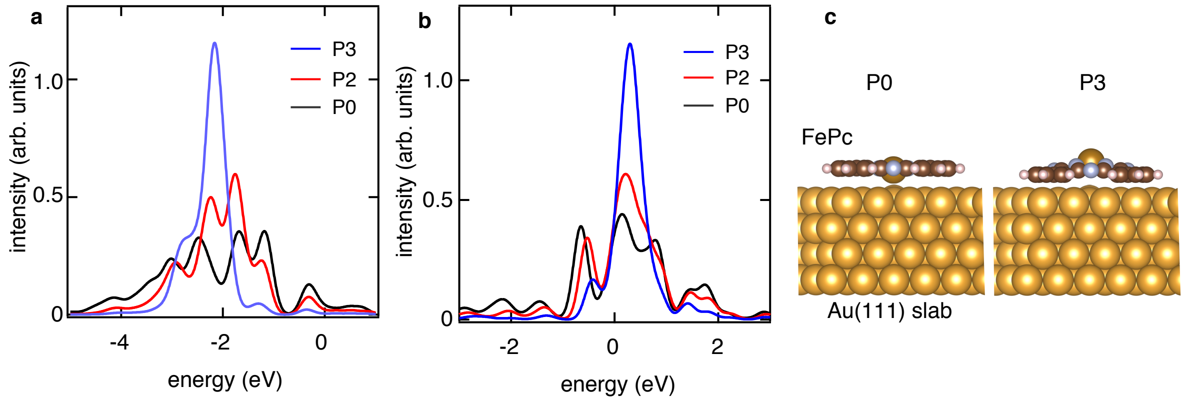

Supplementary Figure 4 | Variation of the d_{z^2}-PDOS with the molecular configuration. (a, b) Variation of the PDOS spectra of **a** majority and **b** minority spin calculated for the molecular configurations of P0, P2 and P3. The calculations are made by using the model structure in which the structure of FePc on Au(111) is fixed as Fig. 3a without the tip. (**c**) Schematic drawings of the molecular configurations for P0 and P3.

Supplementary Note 5 | Evaluation of J_1 and J_2 from the DFT calculations

We evaluate the variation of J_1 and J_2 with the movement of Fe atom. The evaluation was carried out by fitting the PDOS spectra of Fe $3d_{z^2}$ orbital in the P0-P3 configurations shown in Supplementary Fig. 4a with an Anderson-type model Hamiltonian[3,4]. The results of spectral fitting are shown in Supplementary

Fig. 5 together with the parameters listed in Supplementary Table 2. The fitting procedure is described below.

In FePc on Au(111), the Fe $3d_{z^2}$ orbital couples the conduction electrons of both wide *sp*-band and narrow *d*-band of Au(111). Therefore, we used the following Anderson-type model Hamiltonian:

$$H = \sum_{\mathbf{k}\,\sigma} \epsilon_{sp}(\mathbf{k}) c^{\dagger}_{sp\mathbf{k}\sigma} c_{sp\mathbf{k}\sigma} + \sum_{\mathbf{k}\,\sigma} \epsilon_{d}(\mathbf{k}) c^{\dagger}_{d\mathbf{k}\sigma} c_{d\mathbf{k}\sigma} + \sum_{\sigma} \epsilon_a \, c^{\dagger}_{a\sigma} c_{a\sigma} + \sum_{l=sp,d\;\mathbf{k}\sigma} (V_{al\mathbf{k}} c^{\dagger}_{a\sigma} c_{l\mathbf{k}\sigma} + h.c)$$

$$+ U n_{a\sigma} n_{a\bar{\sigma}} \,. (5)$$

Here, $c^{\dagger}_{sp\mathbf{k}\sigma}$ ($c^{\dagger}_{d\mathbf{k}\sigma}$) is the creation operator of conduction electron with spin σ of the *sp*- (*d*-) band, $c^{\dagger}_{a\sigma}$ is that of $3d_{z^2}$ orbital with its energy ϵ_a, $n_{a\sigma}$ is the number operator of the $3d_{z^2}$ orbital, and U is the onsite Coulomb interaction in the $3d_{z^2}$ orbital. Within the Hartree-Fock approximation, the Green's function of the Fe $3d_{z^2}$ orbital can be written as

$$G_{a\sigma}(\omega) = \frac{1}{\omega - \widetilde{\epsilon_a} - \Sigma_{sp}(\omega) - \Sigma_d(\omega)}, (5a)$$

where $\widetilde{\epsilon_a} = \epsilon_a + U < n_{a\bar{\sigma}} >$ and $\Sigma_{sp}(\omega)$ and $\Sigma_d(\omega)$ are the self-energies caused by the hybridization with *sp*- and *d*-bands, respectively. The self-energy is described as

$$\Sigma_l(\omega) = \sum_{\boldsymbol{k}} \frac{|V_{al\mathbf{k}}|^2}{\omega - \epsilon_l(\mathbf{k}) + i\delta} \,. (5b)$$

The subscript l specifies *sp*- or *d*-bands. The local density of states at the Fe atom can be obtained by $\rho_{a\sigma}(\omega) = -\frac{1}{\pi} Im\, G_{a\sigma}(\omega)$. In the spectral fitting, we approximated that the hybridization V_{alk} is independent of the electron wavevector **k**. We also adopted the approximations in the estimation of self-energies as follows: For the *sp*-band, we took the wide-band-limit and approximated as

$$\Sigma_{sp}(\omega) = -i\pi\rho_{sp}(E_F)\left|V_{asp}\right|^2 = -i\Delta_{sp}, (5c)$$

where, $\rho_{sp}(E_F)$ is the density of states of *sp*-band at the Fermi level. For the *d*-band, in order to calculate the self-energy $\Sigma_d(\omega) = \sum_{\mathbf{k}} \frac{|V_{ad}|^2}{\omega - \epsilon_d(\mathbf{k}) + i\delta}$, we described the *d* band of Au(111) as the two-dimensional energy band

$$\epsilon_d(\mathbf{k}) = t\left\{\sum_{\mathbf{k}} 2\cos(k_x) + 4\cos\left(\frac{k_x}{2}\right)\cos\left(\frac{\sqrt{3}}{2}k_y\right)\right\} - M. (5d)$$

Here t is the hopping energy between the nearest-neighboring orbitals and M defines the band center. We focused on the spectral feature around -2 eV in each PDOS spectrum calculated for the configurations of P0, P2 and P3, and fitted the spectrum by using Supplementary Eqs. (5)-(5d).

Supplementary Figure 5 shows the results of spectral fitting. The values of the parameters used are listed in Supplementary Table 2. The spectral features are well reproduced. The fitting results demonstrate

that the d-band is positioned much below E_F (-3 to -2 eV). Thus, the *sp*-band is the main contributor to the hybridization around E_F, and we reasonably exclude the contribution from the d-band to the Kondo Hamiltonian [Supplementary Eq. (4a)]. Since J_1 is proportional to $\frac{4\Delta_{sp}}{\pi\rho U}$, we can evaluate how J_1 and J_2 should vary with the movement of Fe^{2+} ion. As the ion moves away from the Au(111) surface, Δ_{sp} is reduced by 43% in the P2 configuration, and 55 % in the P3 configuration compared to that in the P0 configuration. In the NRG calculations of spectral evolution shown in Fig. 4, we used 40%- and 60%-reduced values for J_1 and J_2 in medium-coupling regime 2 (P2) and weak coupling regime (P3), respectively. The values for J_1 and J_2 in the strong-coupling regime (P0) were taken to reproduce the widths of the Kondo peaks in the high- and low-T_K channels.

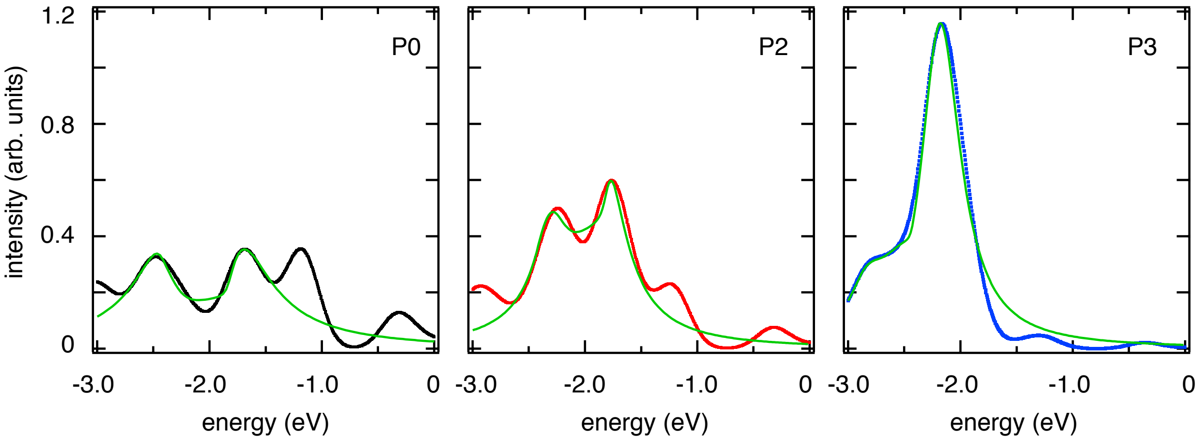

Supplementary Figure 5 | Fitting of the PDOS spectra with an Anderson model. The majority-spin PDOS spectra of d_{z^2} orbital in the configurations of P0, P2 and P3 are fitted with an Anderson model described in Supplementary Note 5. The black, red and blue spectra show the PDOS calculated for P0, P2 and P3. These are the same as those shown in Supplementary Fig. 4a. The green spectra show the fitting results. The parameters used for the fitting are listed in Supplementary Table 2.

Supplementary Table 2 | Parameters used for the spectral fitting. The details about spectral fitting and parameters are described in Supplementary Note 5.

	t (eV)	M (eV)	$\widetilde{\epsilon_a}$ (eV)	Δ_{sp} (eV)	V_{ad} (eV)	δ (eV)
P0	0.1	2.45	-2.05	0.53	0.19	0.05
P2	0.1	2.42	-2.00	0.3	0.11	0.05
P3	0.1	3.05	-2.05	0.24	0.10	0.05

Supplementary Note 6 | NRG calculations of tunneling spectra

In order to explain the evolution of the tunneling spectrum with approaching the STM tip to the Fe^{2+} ion, we calculated the differential conductance ($\mathrm{d}I/\mathrm{d}V$) spectrum. We take into account both elastic and inelastic electron tunneling processes. The former is the normal tunneling and the latter the tunneling with inelastic spin excitation. The elastic process consists mainly of the tunneling through the Kondo resonance appearing as a sharp peak in the DOS spectrum. Thus, the elastic tunneling process provides the DOS spectrum modified with the Fano effect (hereafter denoted as FK resonance spectrum). In the inelastic process, a tunneling electron excites the spin-flip transition between the substates separated by the SOI-splitting renormalized with the Kondo effect. Thus, the $\mathrm{d}I/\mathrm{d}V$ spectrum is described as a linear combination of the FK resonance spectra arising from the channels 1 and 2 and the inelastic spin excitation spectrum. Previous theoretical works reveal that the ratio of inelastic (I_{inel}) to the elastic (I_{el}) current is determined by the exchange coupling of the tunneling electron with the localized spin and the tunneling barrier[5-8]. Actually, the exchange coupling cannot be evaluated quantitatively, and thus the ratio is practically treated as a parameter. We calculate the elastic (I_{el}) and inelastic (I_{inel}) currents independently in the following way and describe the total tunneling current as their linear combination.

The elastic contribution to the $\mathrm{d}I/\mathrm{d}V$ spectrum can be written as

$$\frac{\mathrm{d}I_{\mathrm{el}}(\omega)}{\mathrm{d}V} = C_{\mathrm{ch1}}\sum_{\sigma}\{(q_1^2-1)\Im G_{11\sigma}^{r}(\omega) - 2q_1\Re G_{11\sigma}^{r}(\omega)\}$$

$$+ C_{\mathrm{ch2}}\sum_{\sigma}\{(q_2^2-1)\Im G_{22\sigma}^{r}(\omega) - 2q_2\Re G_{22\sigma}^{r}(\omega)\}. \quad (6)$$

Here, q_1 and q_2 are the constants to determine the Fano lineshapes of the channels 1 and 2, respectively[9]. Note that q_1 and q_2 are not identical to q in Supplementary Eq. (1). The coefficients C_{ch1} and C_{ch2} represent the contributions from the FK resonance spectra of the channels 1 and 2 to the $\mathrm{d}I_{el}/\mathrm{d}V$ spectrum, respectively. The imaginary part of the retarded Green's function in this system is calculated from the definition of the excitation spectrum (DOS spectrum) A_σ,

$$A_\sigma(\omega) = \sum_n \left|< n\left|O_{i\sigma}^{\dagger}\right|gs >\right|^2 \delta(\omega - E_n) + \left|< gs\left|O_{i\sigma}^{\dagger}\right|n >\right|^2 \delta(\omega + E_n)$$

$$= -\frac{1}{\pi}\Im G_{ii\sigma}^{r}(\omega) = -\frac{1}{\pi}\Im \ll O_{i\sigma};\, O_{i\sigma}^{\dagger} \gg. \quad (7)$$

Here, $|gs>$ ($|n>$) represents the electronic ground (excited) state. $O_{i\sigma}$ is the composite operator[10] for spin S_i and defined as

$$O_{i\uparrow} = \frac{J_i}{2}(f_{0i\downarrow}S_i^- + f_{0i\uparrow}S_i^z), \qquad (8)$$

$$O_{i\downarrow} = \frac{J_i}{2}(f_{0i\uparrow}S_i^+ - f_{0i\downarrow}S_i^z), \qquad (9)$$

where J_i is the Kondo coupling constant of the *s-d* interaction for the channel $i = 1, 2$. $f_{0i\sigma}$ is the annihilation operator of the first conduction site in the Wilson chain of the channel i. The real part of the Green's function was obtained by the Kramers-Kronig relation.

The electric current carried by the inelastic electron tunneling process relevant of the spin excitation is defined as

$$I_{\mathrm{inel}} = \frac{1}{4}\sum_m \{< gs|S_-|m >< m|S_+|gs > +< gs|S_+|m >< m|S_-|gs > +< gs|S_z|m > < m|S_z|gs >\}\theta(eV - E_m). \ (10)$$

Here, θ represents the Heviside step function. In the finite $D > 0$, $< gs|S_z|m > < m|S_z|gs >$ is negligibly small so that we can evaluate the inelastic component by

$$I_{\mathrm{inel}} \propto \sum_m \{< gs|S_-|m >< m|S_+|gs > +< gs|S_+|m >< m|S_-|gs >\}\theta(eV - E_m). \ (11)$$

We can calculate the matrix elements $< gs|S_-|m >< m|S_+|gs >$ and $< gs|S_+|m >< m|S_-|gs >$ from the imaginary part of the transverse dynamical magnetic susceptibility, $\chi_{-+}(\omega)$ defined as

$$\Im\,\chi_{-+}(\omega) = \Im\, i\int_0^\infty dt\, e^{i\omega t} < [S_-(t), S_+(t)] > = \sum_{\mathrm{m}} | < m|S_+|gs > |^2\delta(\omega - E_m) - | < gs|S_+|m > |^2\delta(\omega + E_m). \ (12)$$

We calculated the imaginary part of $\chi_{-+}(\omega)$ by NRG, and then calculated the inelastic excitation spectra with Supplementary Eq. (11). In the calculation, we used the same setting as that used in the excitation spectrum calculation.

The DOS spectrum, FK resonance spectrum and the inelastic spin excitation spectrum are calculated with Supplementary Eqs. (7), (6) and (11), respectively. Supplementary Figures 6a and 6b show the DOS spectra calculated for the channels 1 and 2, respectively. In the channel 1, the DOS spectrum in the strong coupling regime (i) shows a peak structure at the Fermi level. This structure is the Kondo resonance arising from the localized electron in d_{z^2} orbital. In the regime (ii), the peak structure is slightly suppressed and a sharp gap appears at the Fermi level. This gap opens as a result of the competition between the Kondo effect and the SOI. In the regime (iii) where J_1 decreases furthermore, the peak structure decays significantly and the gap becomes wider. Finally, in the regime (iv), the Kondo signature

disappears and a residue of the gap remains. Similar variation is observed in the channel 2 (See Supplementary Fig. 6b). Initially, a sharp peak emerges in the regime (i). The peak structure is the Kondo resonance arising from the localized electron in the d_π orbitals. In the regime (ii), the Kondo peak is drastically suppressed and a small gap opens at the Fermi level. This gap also appears as a result of the competition. The Kondo signature is furthermore suppressed from the regime (ii) to regimes (iii) and (iv). The spectra in Fig. 4e are drastically different from those in Supplementary Fig. 6b due to the Fano effect. The peak and gap structures in Supplementary Fig. 6b appear as dip and peak (hump) structures in Fig. 4e. Comparing the decays of Kondo signatures in the channels 1 and 2, the decay in the channel 2 is much faster than that in the channel 1. Thus, we conclude that the spectral evolution observed in the STM experiments mainly comes from the variation of the FK resonance spectrum in the channel 1.

We set $q_1 = 3.0$ and $q_2 = -0.3$ for calculating the FK resonance spectra shown in Fig. 4. These values should change with approaching the STM tip to the Fe^{2+} ion. However, we used the constant values because the variations of the FK resonance spectra (the DOS spectra) and the contribution from the inelastic excitation process are dominant.

Supplementary Figures 6c and 6d show the inelastic spin excitation spectra calculated in the four regimes by the NRG technique. Moving from the regime (i) to the regime (iv), the excitation energy increases, which correlates with the widening of the gap observed in the DOS spectra. As the Kondo couplings decrease with the approach of the tip to the ion, the contribution from the inelastic excitation spectrum enhances, and then the tunneling spectra especially in the regimes (iii) and (iv) look identical to the inelastic spectra. The response of the inelastic spin excitation spectrum to an external magnetic field was also calculated by using Supplementary Eqs. (10)-(12). Supplementary Figure 7 shows the inelastic spin excitation spectra calculated at 0 and 10 T perpendicular to the molecular plane. The spectrum at 10 T consists of the two steps because the step at 0 T splits due to the spin Zeeman effect. These spectra reasonably reproduce the experimental results shown in Fig. 2e, providing a strong support that the inelastic step stems from the excitation between the lower state of $S_z = 0$ and the doubly-degenerate upper states of $S_z = \pm 1$.

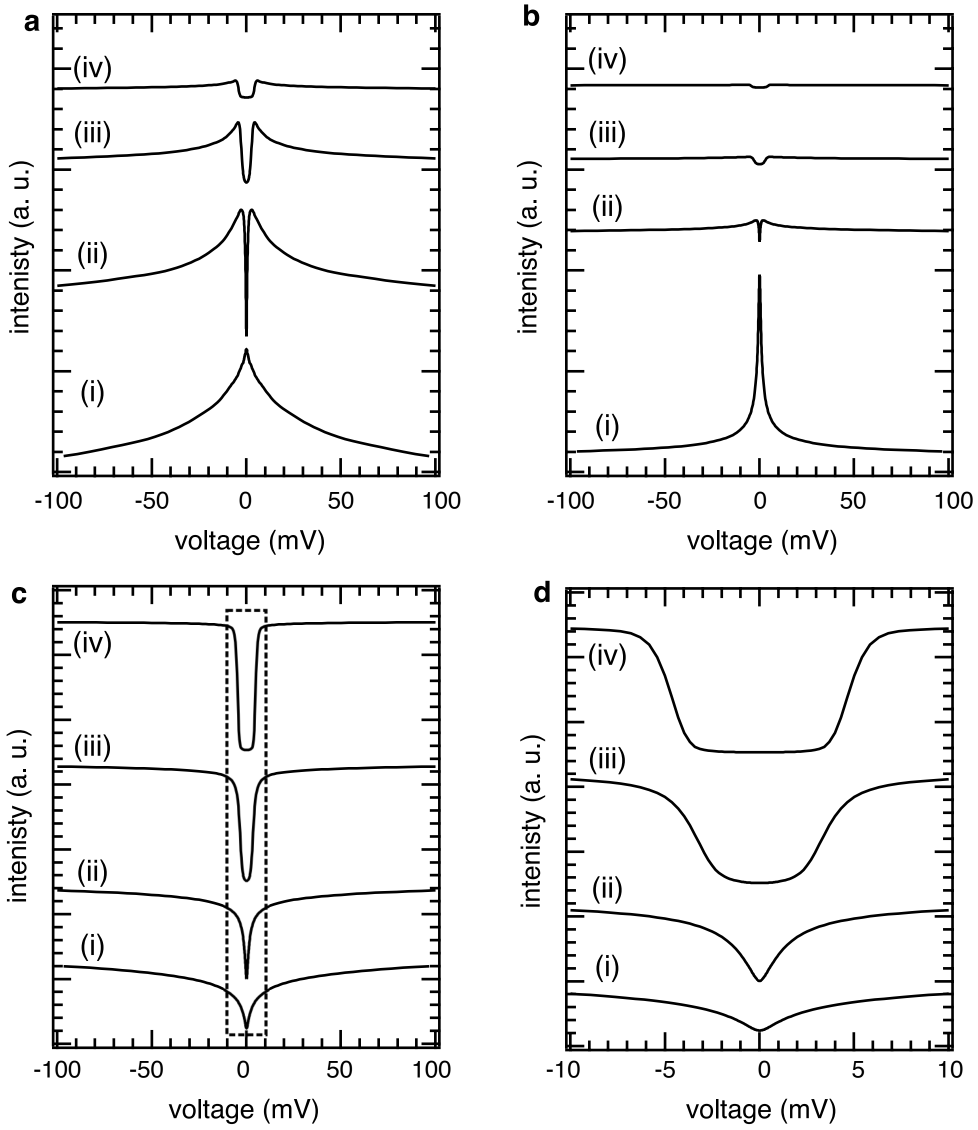

Supplementary Figure 6 | Spectra calculated with NRG technique. (**a**, **b**) DOS spectra calculated in the four regimes for **a** channel 1 and **b** channel 2, respectively. (**c**) Inelastic spin excitation spectra calculated in the four regimes. (**d**) Spectra marked by the dotted rectangle in **c**. The values of J_1 and J_2 used are (i) 0.75 and 0.45 eV, (ii) 0.6 and 0.32 eV, (iii) 0.45 and 0.24 eV and (iv) 0.3 and 0.16 eV, respectively.

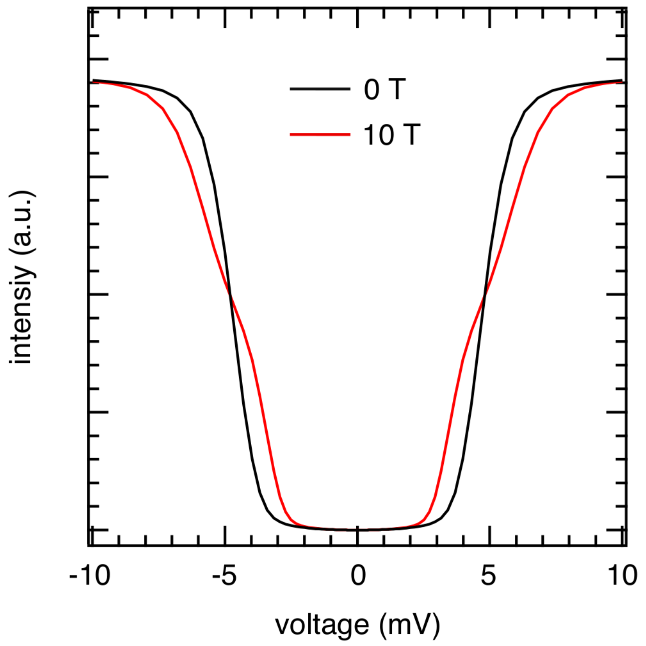

Supplementary Figure 7 | Response of inelastic spectrum to magnetic field. Inelastic spin excitation spectra calculated at 0 (black) and 10 (red) T with NRG technique are shown. The inelastic step at 0 T splits into two steps at 10 T, which reproduces the experimental results.

Supplementary Note 7 | Comparison of Kondo and mixed valence models

The majority- and minority-spin PDOS spectra show that the occupation in the d_{z^2} orbital exceeds 1 and deviates from the half filling configuration suitable to the Kondo Hamiltonian used in the previous. From this feature, one might think that the mixed valence model of the d^6+d^7 configuration[11] better describes the electronic ground state of FePc on Au(111) rather than the Kondo model and that the asymmetric peak in the spectrum A in Fig. 2a is the *d*-resonance. In this section, we discuss the variation of the excitation spectrum caused by the deviation from the half filling, and then we demonstrate that the Kondo model is more reasonable than the mixed valence model.

In order to discuss the influence of the occupation deviation, we consider the following Hamiltonian:

$$H = H_{\mathrm{A}} + H_1, \qquad (13)$$

$$H_{\mathrm{A}} = \sum_{\mathbf{k}\,\sigma\,i=1,2} \varepsilon_{\mathbf{k}i\sigma} c^{\dagger}_{\mathbf{k}i\sigma} c_{\mathbf{k}i\sigma} + \sum_{\sigma\,i=1,2} \left(\frac{U}{2} n_{i\sigma} n_{i\bar{\sigma}} + E_{di} n_{i\sigma} \right) + \sum_{\mathbf{k}\,\sigma\,i=1,2} (V_i\, c^{\dagger}_{i\mathbf{k}\sigma} d_{i\sigma} + h.c.\,), (13a)$$

$$H_1 = J_{\mathrm{H}} \mathbf{S}_1 \cdot \mathbf{S}_2 + D \left(\sum_{i=1,2} S_{iz} \right)^2. \quad (13b)$$

H_{A} is two-orbital Anderson Hamiltonian. The first term of Supplementary Eq. (13a) represents the Au(111) surface electron. The second term indicates the occupation-dependent effective energies of d_{z^2} and d_{π} orbitals where U is the intra-orbital Coulomb repulsion and $n_{i\sigma} = d^{\dagger}_{i\sigma} d_{i\sigma}$ is the occupation in each orbital with the creation ($d^{\dagger}_{i\sigma}$) and annihilation ($d_{i\sigma}$) operators at the d_{z^2} ($i = 1$) and d_{π} ($i = 2$) orbitals. The third term indicates the hybridizations of these orbitals with the surface electrons. Supplementary Eq. (13b) is identical to Supplementary Eq. (4b). The definitions of the operators, constants and the indexes are the same as those in Supplementary Eqs. (4a) and (4b). As typical values, here we set $J_{\mathrm{H}} = -\frac{U}{2}$ and D = 5 meV.

The effective energies of the d_{z^2} orbital are written as E_{d1} and $E_{d1} + U$ in the limit of $V_1 = 0$ from Supplementary Eq. (13a). The Hamiltonian effectively results in the Kondo Hamiltonian by assuming sufficiently large U and the particle-hole symmetry ($E_{d1} = -U/2$). By varying E_{d1} from $-U/2$ to $-U$, the Hamiltonian describes the variation from symmetric Anderson model to asymmetric Anderson and the mixed valence models as shown in Supplementary Fig. 8. The typical excitation spectra of these three models are well known[12]. In the symmetric Anderson model ($E_{d1} = -U/2$), a symmetric Kondo peak appears at the Fermi level with a pair of side peaks at $\pm U/2$. In the asymmetric Anderson model ($-\frac{U}{2} < E_{d1} < -U$), the Kondo peak becomes slightly asymmetric. Instead of the Kondo peak, in the mixed valence model ($E_{d1} = -U$) appears an asymmetric broad peak stemming from the charge fluctuation in the mixed valence configuration.

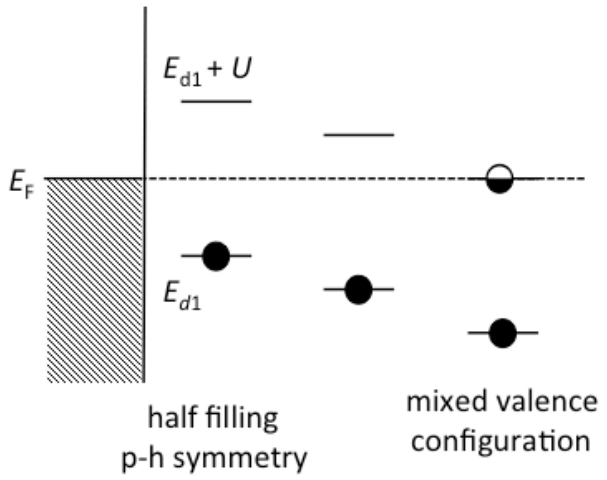

Supplementary Figure 8 | Energy diagrams of d_{z^2} orbital. In the limit of $V_1 = 0$, two d_{z^2} levels are evolved from a symmetric Anderson regime to an asymmetric one with the variation of E_{d1}. For the nonzero V_1, these levels get broadened due to the hybridization with the conduction electrons and a peak appears at the Fermi level as a result of Kondo effect or the charge fluctuation in the mixed valence configuration.

We calculated the variation of the excitation spectra by using the NRG method with Supplementary Eqs. (13), (13a) and (13b) for $E_{d1} = -0.75U, -0.8\,U$ and $-U$. In the calculations, we set $E_{d2} = -U/2$ and $U = 1.0$ eV for both orbitals. In addition, we used $\Delta_i = \pi\rho V_i^2$ $(i = 1, 2)$ as parameters to describe the hybridization strengths of both orbitals with the conduction electrons. The larger (smaller) Δ_i indicates the stronger (weaker) hybridization and results in the Kondo (SOI)-dominant regime.

Supplementary Figure 9 shows the calculated excitation spectra. The spectra in Supplementary Figs. 9a, 9c and 9e correspond to the Kondo (or mixed valence) regime while the spectra in Supplementary Figs. 9b, 9d and 9f to the SOI-dominant regime. One sees a sharp peak at the Fermi level in the spectrum of channel 1 (d_{z^2} orbital) in Supplementary Fig. 9a. A similar peak appears in Supplementary Fig. 9c. These peaks originated from the Kondo effect. In contrast, the spectrum is drastically changed at $E_{d1} = -U$ as shown in Supplementary Fig. 9e; a broad asymmetric peak appears below the Fermi level. This broad peak originates from the charge fluctuation in the mixed valence configuration. The spectra of channel 2 (d_π orbital) are hardly changed as shown in Supplementary Figs. 9a, 9c and 9e, and each sharp peak is the Kondo resonance state.

The spectra in the SOI-dominant regime show a dip structure around the Fermi level in the channel 1 as shown in Supplementary Figs. 9b, 9d and 9f. The dip arises from the energy gap between the $S_z = 0$

and $S_z = \pm 1$ states formed by the SOI. Each spectral shape is the convolution of the Kondo peak (or charge fluctuation peak) and the dip. Comparing the spectral intensity outside the gap in Supplementary Fig. 9a with the counterpart in Supplementary Fig. 9c, the intensity is reduced drastically. The same is true for Supplementary Figs. 9b and 9d. The reduction stems from the suppression of the Kondo effect by the SOI. The reduction of the Kondo peak is also observed more clearly in the channel 2 where the half filling is satisfied. Thus, the drastic reduction is characteristic response of the Kondo resonance state to the SOI in this model. In contrast, when comparing the spectra in Supplementary Figs. 9c and 9f, the peak associated with the charge fluctuation is not reduced in intensity in the SOI-dominant regime but instead it is enhanced. This is because the charge fluctuation does not compete with the SOI and the decrease of Δ_1 makes the peak sharper.

The spectral variation derived from the SOI discussed above enables us to solve the problem which model better describes the electronic ground state of FePc on Au(111), Kondo or mixed valence model. We conclude that the Kondo model is more reasonable for the electronic ground state of FePc on Au(111). As shown in Fig. 2a, the asymmetric peak observed when the tip is far away from the molecule disappears by approaching the tip to the molecule. This spectral variation reasonably matches with that calculated in the Kondo model. However, the mixed valence model cannot explain the variation even if the broad asymmetric peak in the mixed valence model reasonably explains the spectrum signature measured experimentally when the tip is far away. Based on these results, we rule out the possibility that the electronic ground state is the mixed valence configuration of $d^6 + d^7$, and we justify our analysis with the Kondo model. In the NRG calculations discussed in the main text, we used a simplified version of Supplementary Eq. (13), i.e., Supplementary Eq. (4), to reduce the number of parameters and extract the essential physics behind the spectral evolution found in the STM manipulation experiments.

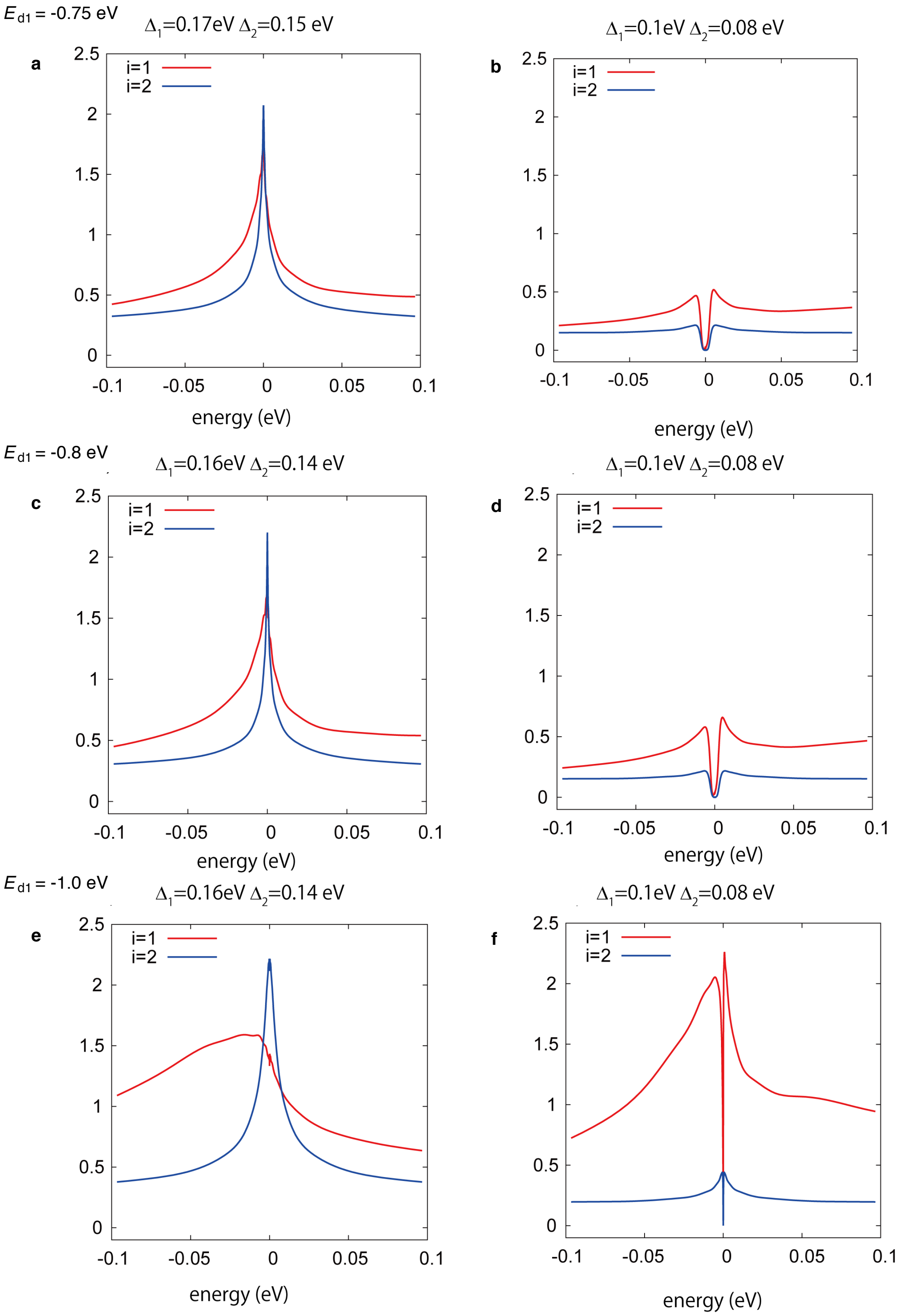

Supplementary Figure 9 | Calculated excitation spectra. (**a**, **c** and **e**) show the spectra in the Kondo (or mixed valence) regime while (**b**, **d** and **f**) those in the SOI-dominant regime. The calculations are carried out as functions of E_{d1}, Δ_1 and Δ_2. Red and blue curves show the excitation spectra calculated for channels 1 and 2, respectively.

Supplementary References

1. Fano, U. Effects of configuration interaction on intensities and phase shifts. *Phys. Rev.* **124**, 1866-1878 (1961).
2. Lambe, J. & Jaklevic, R. C. Molecular vibration spectra by inelastic electron tunneling. *Phys. Rev.* **165**, 821-832 (1968).
3. Lucignano, P., Mazzarello, R., Smogunov, A., Fabrizio, M. & Tosatti, E. Kondo conductance in an atomic nanocontact from first principles. *Nat. Mater.* **8**, 563-567 (2009).
4. Requist, R., Modesti, S., Baruselli, P. P., Smogunov, A., Fabrizio, M. & Tosatti, E. Kondo conductance across the smallest spin 1/2 radical molecule. *Proc. Natl. Acad. Sci.* **111**, 69-74 (2014).
5. Nussinov, Z. Crommie, M. F. & Balatsky, A. V. Noise spectroscopy of a single spin with spin-polarized STM. *Phys. Rev. B* **68**, 085402 (2003).
6. Fransson, J. Spin inelastic electron tunneling spectroscopy on local spin adsorbed on surface. *Nano Lett.* **9**, 2414-2417 (2009).
7. Fransson, J., Eriksson, O. & Balatsky, A. V. Theory of spin-polarized scanning tunneling microscopy applied to local spins, *Phys. Rev. B* **81**, 115454 (2010).
8. Delgado, F. & Fernández-Rossier, J. Spin dynamics of current-driven single magnetic adatoms and molecules. *Phys. Rev. B* **82**, 134414 (2010).
9. Újsághy, O., Kroha, J., Szunyogh, L. & Zawadowski, A. Theory of the Fano resonance in the STM tunneling density of states due to a single Kondo impurity. *Phys. Rev. Lett.* **85**, 2557-2560 (2000).
10. Costi, T. A. Kondo effect in a magnetic field and the magnetoresistivity of Kondo alloys. *Phys. Rev. Lett.* **85**, 1504-1507 (2000).
11. Stepanow, S., Miedema, P. S., Mugarza, A., Ceballos, G., Moras, P., Cezar, J. C., Carbone, C., de Groot, F. M. F. & Gambardella, P. Mixed-valence behavior and strong correlation effects of metal phthalocyanines adsorbed on metals, *Phys. Rev. B* **83**, 220401 (2011).
12. Hewson, A. C. *The Kondo problem to heavy fermions* (Cambridge Univ. Press, Cambridge 1993).
